# Attrition in a large‐scale habituation task administered at home

**DOI:** 10.1111/bjdp.12528

**Published:** 2024-10-25

**Authors:** Maximilian Seitz, Dave Möwisch, Manja Attig

**Affiliations:** ^1^ Leibniz Institute for Educational Trajectories Bamberg Germany

**Keywords:** attrition, habituation, panel study

## Abstract

Infant research often struggles with selective samples, especially when focusing on behavioural measures, such as those drawn from habituation tasks. However, selectivity may threaten the generalizability and interpretation of results, which is why the current study investigates attrition in a habituation task administered in a household setting in 7‐month‐old infants. We used a large‐scale German dataset, focusing on the children's socioeconomic background, and investigated two aspects of attrition, namely, participation and task completion. The findings suggest significant effects of the children's socioeconomic background on attrition: Maternal education, parental occupation, household income and household language (German vs. other) were positively related to participation and task completion. The analyses indicate that multiple barriers may prevent parents from lower socioeconomic backgrounds from letting their children participate. The study concludes with a critical discussion of possible mechanisms of selectivity in behavioural measures as well as the household setting, in which the data were collected.

**Statement of Contribution**

**What is already known on the subject:**

- Sample selectivity, for example regarding socioeconomic background, threatens the generalizability and interpretation of results.
- Infant research often struggles with selective samples and information on attrition in habituation tasks is scarce.

**What the present study adds:**

- Large‐scale data is used to investigate associations between children’s socioeconomic background and attrition in a habituation task.
- Initial participation (i.e., informed consent) and task completion as distinct indicators of attrition are examined.

## BACKGROUND

In the last decades, tasks assessing habituation and dishabituation as indicators of non‐associative learning have become a standard method for tapping into the cognitive abilities of young children (Colombo et al., 2020). However, there is a recent debate over the internal and external validity as well as the reproducibility of results from studies using habituation tasks – issues that also concern other behavioural paradigms in infant and child development research (Byers‐Heinlein et al., 2022; Singh et al., 2023). The validity of research findings is partly criticized because of attrition, which is a central issue in infant research (among other disciplines), especially in experimental designs that require direct behavioural responses from the child (Slaughter & Suddendorf, 2007). Methodologically, it can be reasoned that attrition may bias what kind of data are available, resulting in issues regarding statistical power and generalization (Dworksy, 2014). Infant research is often realized in laboratories focusing on selective samples (Bornstein et al., 2013) that provide limited means of specifically addressing mechanisms of attrition (Oakes, 2017). In addition, habituation tasks have traditionally been rare in large‐scale studies, which have the advantage of more heterogeneous samples and larger sample sizes (Hachul et al., 2019). Therefore, the current study aims to extend this line of research by using data from a German large‐scale panel study to investigate predictors and characteristics of attrition in habituation tasks, focusing on children's socioeconomic background.

### Theoretical background

Attrition, subject loss, drop‐out, non‐response, and non‐completion collectively refer to the same phenomenon and are sometimes used interchangeably in the social sciences (Hartmann, 2005); some authors also suggest using subject mortality or sample retention in longitudinal designs (Barry, 2005; Dworksy, 2014). These terms are about how a participant's decision leads to non‐participation or premature termination of their involvement in a data collection program before its completion. Thus, these terms both concern the involvement in the context of a single experiment or survey and in future experiments or surveys (i.e., longitudinal designs), in which data are collected. While there are suggestions to differentiate between these terms, such differentiations are rarely consistent (Pulkkinen & Kokko, 2012). At a most basic level in cross‐sectional designs, it was suggested to differentiate between subjects who refuse *participation*, which can be seen as individuals of a gross sample not entering the net sample, and subjects who participate in but do not *complete* a study (Eysenbach, 2005). Consequently, we focus on participation and task completion as indicators of attrition in habituation tasks. This terminology is typically used in survey research with adults but attrition in infants and young children should be even more critical because of the limited means of self‐regulation as another person – parents – decide about participation and well‐being during the tasks (Jungmann et al., 2023).

Historically, survey research has been investigating how sampling strategies may influence the outcomes of scientific investigations (Krosnick, 1999). From a methodological perspective, this research line investigates how to explain attrition, find differential indicators for measuring attrition, anticipate any potential bias in the data and statistically correct for it (Olsen, 2005) – typically with a focus on the stability, heterogeneity and representativeness of samples in longitudinal panel surveys based on probability sampling (Glaser, 2012). In surveys, attrition rates may vary across periods, data sources and outcomes, due to, for example, refusal to participate, inability to locate the participants, dropping out of the study or inconsistent data (Dworksy, 2014). While participation and task completion can have situational (i.e., random) reasons, non‐random and systematic attrition may threaten the internal and external validity of the data, the statistical power, as well as the generalizability of the findings (De Leeuw & Lugtig, 2015). Possible consequences are attrition bias or non‐response bias, which refers to how non‐participation and non‐completion may result in selectivity of sample characteristics (Little & Rubin, 1989). In other words, such biased data limit the interpretation of the results, which is why theoretical frameworks from survey research are also important for developmental sciences. While there are statistical approaches to counter attrition bias, such as weighting and multiple imputation (Nicholson et al., 2017), these approaches are of limited use in understanding the mechanisms behind attrition.

The perspective of survey research on attrition draws on theoretical frameworks of survey response behaviour and cooperation. Participation and task completion can be explained by decisional processes that iterate between stages of postponement, rejection and ultimately non‐response (Glaser, 2012). In other words, people constantly rationalize about participation, and this is associated with the perceived (or expected) efforts and benefits of providing cooperation in a survey. Three types of factors influence whether potential participants cooperate and provide complete data, namely, external factors (i.e., exogenous variables not directly related to survey or person characteristics, such as the overall survey‐taking climate or urbanicity effects), survey‐related factors (e.g., design, size and topic) and internal factors (i.e., person characteristics) (Albaum & Smith, 2012). While this framework lists decisions for one's participation, similar – and possibly even stricter – mechanisms are to be expected for decisions for the participation of one's children (Jungmann et al., 2023; Mauthner, 1997).

The current study primarily investigates the internal factors of decision‐making. It should be noted that regarding infant research, internal factors may refer to both the infants as well as the caretaker. It is a complex network of bidirectional processes that influence both participation and task completion; that means, characteristics of the caretaker as well as (perceived) characteristics of the infant might directly or indirectly lead to task non‐completion or non‐participation. The current study primarily focuses on person characteristics of the caretaker, namely, the socioeconomic background that influences the decision‐making process (Lee, 2003). Socio‐emotional characteristics and psychological predispositions in both infants and caretakers are not examined (Albaum & Smith, 2012). While this framework mainly draws on survey methodology, its theoretical and practical implications are also relevant for infant observations in developmental psychology (Bornstein et al., 2013; Nicholson et al., 2017).

### Attrition in infant research

Infant research is notoriously challenging due to several barriers regarding sample recruitment and the observation or testing of young children (Oakes, 2017). Indeed, a recent discussion on the reproducibility and generalizability of infant research highlights that previous research often lacked statistical power and sufficiently heterogeneous samples (DeBolt et al., 2020; Singh et al., 2023) – both of these issues are associated with attrition (Little & Rubin, 1989). While there are findings that suggest non‐systematic attrition in behavioural measures (Klein‐Radukic & Zmyj, 2015; Segal et al., 2021), there are numerous reasons for potentially systematic attrition (i.e., participation and task completion) such as child factors (e.g., health or temperament), parental factors (e.g., socioeconomic background or personality), technical factors (e.g., experimenter or setup error) and external factors (e.g., interruptions by a third party).

While previous research focused on child factors (Klein‐Radukic & Zmyj, 2015; Segal et al., 2021; Slaughter & Suddendorf, 2007), the current study investigates parental factors that are central to the data collection process and have often been overlooked in the literature. In other words, young children are unable to provide informed consent, which is why parental factors are essential for participation. In addition, parents also have significant control over later stages in the data collection process (Jungmann et al., 2023; Mauthner, 1997). They may either choose to not let their child participate or withdraw informed consent during or after participation due to personal reasons (e.g., limited resources or data security concerns) or if they perceive any discomfort or harm to their child. It was shown that socioeconomic background is negatively associated with attrition in surveys in adult samples (Lee, 2003) – comparable findings from infant research are rare due to the typically small and selective samples (Oakes, 2017).

It can be reasoned that behavioural measures, such as those drawn from habituation tasks, generate a considerable attrition rate because the parents refuse participation or task completion. A review of 101 studies using habituation and violation of expectation tasks published between 1985 and 2005 found that the mean level of attrition due to task non‐completion was modest, although there was a large variance between studies (*M* = 13.70%, Min = 0%, Max = 62%) (Slaughter & Suddendorf, 2007). The authors focused on maladaptive aspects of the infants' state (i.e., fussiness, crying or drowsiness), and they exclusively drew on small‐scale laboratory research, which means that there is no information on parents who are not willing to participate in the experiments. While sometimes there is information on child factors that lead to task non‐completion, parental factors that lead to a lower rate of participation cannot be analysed in this way.

In large‐scale panel studies, this may be different. Here, an extensive amount of data is collected on a typically heterogeneous sample (Davis‐Kean & Jager, 2012). In addition, survey participants may need to give additional informed consent to tasks involving their children (e.g., due to stricter data security regulations). Therefore, large‐scale studies often provide information on parents and their children even when they do not participate in any specific task. It is important to note that there is still a certain amount of selectivity to be expected because of the discrepancy between the gross and net sample. In addition, non‐participation in such settings can partly be explained by situational factors (Albaum & Smith, 2012) that should, however, occur at random and are not the focus of the current study (e.g., interruptions from the standardized procedure). Still, large‐scale studies could provide a certain insight into the mechanisms of participation and task completion due to the large amount of data that is collected about the children and their socioeconomic background (Jungmann et al., 2023).

To the knowledge of the authors, habituation tasks administered in a large‐scale context are rare. The Avon Longitudinal Study of Parents and Children (ALSPAC) was a British population‐based study that started data collection in 1991 (The ALSPAC Study Team, 2019). In ALSPAC, a habituation task was administered in 4‐month‐old infants (range 3.75–5.25 months) in a randomly drawn subsample of about 10% of the overall cohort (Moulton et al., 2020). Regarding task completion, girls and younger infants were more likely to not complete the habituation task but parental socioeconomic background was not further explored (Bell et al., 1998). Preterm birth status, often associated with distinct habituation patterns (Kavšek & Bornstein, 2010) and an increased probability of attrition (Teixeira et al., 2021), was not analysed similarly. Habituation tasks were also administered at 3 months in the Mannheim Study of Children at Risk (MARS; Esser & Schmidt, 2017) and between 3 and 9 months in the Kansas Early Cognition Project (Colombo et al., 2004) but there are no systematic attrition analyses available.

More recently, the ‘ManyBabies’ project offers exciting opportunities for investigating developmental phenomena from a large‐scale, cross‐cultural perspective (Visser et al., 2022). Attrition analyses are still rare possibly due to the ongoing data collection. Steffan et al. (2024), for example, investigated attrition in assessing infants' goal‐directed anticipatory‐looking behaviour but did not analyse aspects beyond the assessment methodology. Finally, the Newborn Cohort of the German National Educational Panel Study (NEPS SC1; Blossfeld & Roßbach, 2019) featured habituation tasks at 7 and 17 months. The longitudinal panel study is carried out by a nationwide interdisciplinary scientific network of researchers and focuses on education, which is why early cognitive abilities as precursors of later skills and competencies have been assessed since the first year of life (Attig et al., 2023).

### The current study

Taken together, selectivity due to attrition can bias sample characteristics, which is a prominent issue in infant research (Oakes, 2017; Singh et al., 2023). Parental factors (e.g., socioeconomic background) potentially contribute to attrition in behavioural research designs (Jungmann et al., 2023), such as habituation tasks. Theoretical frameworks model attrition as a complex, decision‐stage process (Glaser, 2012), highlighting that parental factors may contribute to sample selectivity by influencing participation as well as task completion during the data collection process (Albaum & Smith, 2012). While participation should have many causes that also relate to aspects of the survey, parental factors might explain a certain amount of selectivity. However, regarding habituation tasks, previous research is often limited to analysing task completion in small laboratory samples, and predictors of participation can rarely be examined. Therefore, we used data from a German large‐scale panel study to investigate participation and task completion in a habituation task administered at 7 months in the households of the families.

More specifically, we were interested in the relation between participation, task completion and parents' socioeconomic background. Drawing on findings from surveys (Lee, 2003), we expected that parents from low socioeconomic backgrounds are less likely to give informed consent to participating in the habituation task. In addition, we provide information on the reasons parents reported for not letting their child participate in the habituation task. Regarding task completion, we expected that children from low socioeconomic backgrounds are less likely to complete the task due to parental interference (Jungmann et al., 2023) or environmental aspects detrimental to the task administration (Evans et al., 2010). Finally, we consider a stricter approach towards task completion in an exploratory analysis because not every reported task completion resulted in available looking data (see below).

## METHOD

### Sample

The current study used data collected in NEPS SC1 (Attig et al., 2023; NEPS Network, 2021). NEPS is an interdisciplinary panel study that investigates educational processes using a multi‐stage cohort design (for more information, see Blossfeld & Roßbach, 2019). NEPS SC1 is a representatively drawn sample of infants born between February and June 2012 in Germany (gross sample *N* = 8483; for more information, see Würbach et al., 2016; Zinn et al., 2020). NEPS SC1 started (i.e., newborn children as target persons) when the children were on average 7 months old (*M* = 7.00 months, *SD* = 0.76, Min = 5.15, Max = 11.93; 50.99% male, 49.01% female). Data are only available for people who agreed to participate in the survey (net sample *N* = 3481). In the first step, this sample was used for investigating participation in the habituation task. In the second step, a subsample was used to investigate task completion. For this subsample, we considered all cases with complete video recordings of the habituation task (*N* = 2945). Finally, for the stricter approach in analysing only those cases with available looking data, we used a subsample considering all cases with complete video recordings without disturbances as well as complete codings from the videos (*N* = 2235).

### Assessment of the habituation task

When the children were on average 7 months old, two categorical fixed‐trial habituation subtasks were administered back‐to‐back (total presentation time: 6.50 min), which will be analysed collectively in the context of the current study. For all children, the two subtasks were presented in the same sequence, and they were embedded in a larger battery of observation and interaction tasks (total time: 18.50 min), for which informed consent was asked independent of the habituation task (Attig et al., 2023). Trained interviewers administered the task in the children's homes, which is why a fixed‐trial procedure was chosen to achieve standardization, given the heterogeneous household conditions (Weinert et al., 2016). Aspects that were identified as essential in the training were correct instructions for the parents, adequate interaction behaviour with the children, sufficient lighting and furnishing, the laptop setup and the camera settings.

The stimulus material was categorical, so each trial featured one yellow, round‐shaped flower (first subtask) or one bug in various colours (second subtask) respectively. The dishabituation phase featured one blue, rectangular flower (first subtask) or one blue, rectangular bug (second subtask) respectively. The presentation time was 10 (habituation phase) or 15 s (dishabituation phase) per trial, accumulating to a total time of 355 s for both subtasks (i.e., 13 trials). Examples of the stimulus material can be found in the Supporting Information (Table S4). The children's looking behaviour was recorded on video and manually coded off‐line by independent raters (unadjusted level of agreement: 95%; *ĸ* = .92). The raters were also responsible for judging whether the video recording could be validly coded, namely, whether the video quality allowed for determining the looking behaviour. Therefore, frequent or severe technical, external (i.e., interruptions from the standardized procedure), or child‐related disturbances could result in the data not being released. More detailed information on the task administration and the stimulus material can be found in the official documentation (Seitz et al., 2023).

### Indicators of attrition

We used two primary indicators and one additional exploratory indicator of attrition in the habituation task for the current study: Participation (i.e., informed consent) and task completion.

#### Participation

When participating in NEPS SC1, namely, being part of the net sample, parents were interviewed at home by a trained interviewer. The parents needed to give additional informed consent for their child to participate in the habituation task due to the need for video recordings, covering both subtasks (0 = Non‐participation; 1 = Participation). This consent also included having their child video recorded during the task.

#### Task completion

The second indicator refers to children who completed both habitation subtasks (0 = Task non‐completion; 1 = Task completion). Thus, task completion covers cases in which both habituation subtasks were administered, and a video recording could be retrieved.

#### Valid looking data

It is important to note that not in all cases, in which a video recording was available, looking data could be coded. Cases with severe or multiple technical, external or child‐related disturbances were marked as inadequate during the coding process. For these cases, no looking data could be coded (Seitz et al., 2023). For example, the video coders screened the recording for video quality, namely, whether the infants' eyes could be clearly seen. When this was not the case, for example, due to poor resolution, lighting or fussiness, the video was not coded, and the looking data were not released. Therefore, we analysed the discrepancy between the available video recordings and cases with published looking data (0 = No valid looking data available; 1 = Valid data available). We chose this additional exploratory approach keeping in mind that we could not differentiate between technical, external or child‐related issues that lead to ‘non‐valid’ cases.

### Parental factors

Regarding children's socioeconomic background, we included various aspects of parental socioeconomic status (SES) (Duncan & Magnuson, 2012). More specifically, we used maternal education, parental occupation, household income and household language to define a latent factor of parental SES at 7 months. We included maternal education (i.e., years of education), as in most cases the biological mother gave informed consent in the habituation tasks (Seitz et al., 2023). Regarding parental occupation, we used the highest International Socio‐Economic Index of Occupational Status (HISEI) in the household. The measure considers the required education level and the mean income of a specific occupation to create a continuous classification scheme and is a standard index for social stratification (Ganzeboom et al., 1992). For household income, we used the standard OECD (2013) approach of calculating a household‐size adjusted score that weights people below and above the age of 14 differentially (i.e., equivalized household income). We standardized the variable to account for outliers. Finally, we used a dichotomous indicator of the children's household language as another family characteristic (0 = not only German; 1 = only German) (Diemer et al., 2013). The structural model showed good model fit: *χ* ^2^(2) = 32.68, *p* < .01; CLI = .988, RMSEA = .07, SRMR = .02.

### Control variables

The focus of the current study was on parental factors associated with attrition, namely, the intentional decision to let their child participate, which is why infant‐related characteristics were only included as control variables. While some findings suggest that girls may show a higher attrition rate (Wachs & Smitherman, 1985), a review on task completion could not confirm this (Slaughter & Suddendorf, 2007). Younger children, however, may show higher non‐completion rates (Bell et al., 1998). Regarding participation, age and gender may play a role in clinical studies (Jungmann et al., 2023) but we had no reason to believe that it may influence parents' consent. Therefore, we only included age and gender as control variables. Children's age was also included due to the broad age range when compared to typical laboratory studies administering habituation tasks (*M* = 7.00 months, *SD* = 0.76, Min = 5.15, Max = 11.93). For children's gender, we used a dichotomous indicator of the children's biological sex assigned at birth, as reported by the parents (50.99% male, 49.01% female). Finally, children's preterm birth status (<37 weeks of gestation; 8.39%) was included as another control variable that was identified as having a higher risk of attrition in studies involving experimental paradigms (Teixeira et al., 2021).

### Statistical analyses

We used structural equation modelling to analyse participation and task completion as binary dependent variables in two separate models. In addition, we provide information on an exploratory model focusing on the discrepancy between task completion and valid looking data. For all models, we use R 4.2.2 (The R Foundation for Statistical Computing, 2022) (package lavaan 0.6‐16; Rosseel, 2012), whereas data curation and more basic calculations were done in Stata 17.0 (StataCorp, 2021). In the models, we included the control variables (i.e., age, gender and preterm birth status) as manifest variables. The four aspects of the children's socioeconomic background (i.e., maternal education, HISEI, household income and household language) were included as latent factor of parental SES because the theoretical framework does not provide an adequate basis for assuming specific mechanisms of attrition. There were several missing values regarding most independent and control variables (Table 2 and Table S1). We used full information maximum likelihood to estimate these missing values (Lee & Shi, 2021).

## RESULTS

Table 1 provides a descriptive overview of participation, task completion and cases with valid looking data. Table 2 provides descriptive information on all independent and control variables used for analysing participation (net sample of NEPS SC1); information on the subsamples on task completion (Table S1) and valid looking data (Table S2) can be found in the Supporting Information. Information on bivariate correlations can also be found in the Supporting Information (Table S3). The participating subsample was younger than non‐participants, *t*(3472) = 4.14, *p* < .01, *d* = 0.23, had higher levels of maternal education, *t*(3412) = 8.98, *p* < .01, *d* = 0.52, had parents with higher household income, *t*(3366) = 6.60, *p* < .01, *d* = 0.38 and occupation level, *t*(2976) = 8.13, *p* < .01, *d* = 0.51 and German as their only household language more often, *t*(3479) = 6.54, *p* < .01, *d* = 0.37.

**TABLE 1:** Overview of subsamples for analysing attrition.

Attrition variable	Observations
Participation	Participation *N* = 3129; non‐participation *N* = 352 (Total: *N* = 3481)
Task completion	Task completion *N* = 2945; non‐completion *N* = 184 (Total: *N* = 3129)
Valid looking data	Valid data *N* = 2354; no valid data *N* = 591 (Total: *N* = 2945)

*Note*: Overall net sample size for NEPS SC1 at 7 months *N* = 3481; valid looking data excludes cases with inadequate coding due to technical, external or child‐related reasons.

**TABLE 2:** Participation: descriptive overview of predictors and control variables.

Variable	Participation (*N* = 3129)		Non‐participation (*N* = 352)	
	*M* (*SD*)	Range	*M* (*SD*)	Range
Education (years)	14.66 (2.60)	9–18	13.30 (2.84)	9–18
Household income (€)	1652.46 (897.79)	95.24–15,555.56	1316.25 (771.37)	244.90–5714.29
HISEI	63.07 (19.75)	12.01–88.96	52.83 (22.58)	11.74–88.70
Household language	447 (14.29%) not only German; 2682 (85.71%) German		97 (27.56%) not only German; 255 (72.44%) German	
Age (months)	6.98 (0.74)	5.77–11.93	7.16 (0.91)	5.15–11.74
Gender	1597 male (51.04%); 1532 female (48.96%)		178 male (50.57%); 174 female (49.43%)	
Preterm	2874 full‐term (91.85%); 255 preterm (8.15%)		315 full‐term (89.49%); 37 preterm (10.51%)	

*Note*: Missing values: Age (0.20%); education (1.92%); household income (3.25%); HISEI (14.45%); gender (0%), preterm (0%); household language (0%); an overview for the subsamples on task completion (Table S1) and valid looking data (Table S2) can be found in the Supporting Information.

In addition, the respondents were asked why they refused to let their child participate in the habituation task (non‐participation; Table 3). This list is based on a question to the respondent after participation refusal that provided predetermined categories that were identified in previous feasibility studies. A large proportion of respondents did not state their motivation (42.90%). However, when parents reported a reason to refuse participation, it was mostly due to the requirement of filming during task administration, possibly linked to data security concerns and having no adequate furniture for the experimental setup, which was mainly a table of adequate height. Only a few parents reported feeling unwell or that their child was unwell for task administration (i.e., health‐related reasons).

**TABLE 3:** Descriptive overview of self‐reported reasons to refuse participation.

Reason for refusal	Observations
No adequate furniture for experimental setup	82
Filming	70
Data security concerns	20
Child reasons (unwell)	17
Laptop for experimental setup not allowed	10
Parental reasons (unwell)	5
Other reasons	40
No answer given	151

*Note*: *N* = 352 refusals; respondents could name multiple reasons.

**FIGURE 1:**
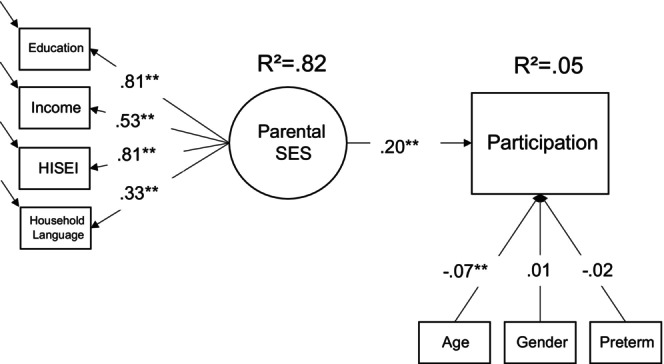
Model predicting participation in the habituation task. *N* = 3481 (FIML), *χ* ^2^(17) = 57.30, *p* < .01. CLI = .986, RMSEA = .03, SRMR = .02 (standardized coefficients reported); *p < .05, **p < .01. Coding: 0 = Non‐participation; 1 = Participation.

For analysing participation, we used the whole net sample of NEPS SC1 (*N* = 3481); *N* = 3129 gave informed consent in the habituation task. Figure 1 shows that children's socioeconomic background, namely, parental SES, is positively associated with participation in the habituation task (see also Table 2 and Table S1). Concerning the control variables, both preterm birth status and children's gender do not have a significant effect on participation, while parents of younger children were more likely to let them participate. Overall, the model explains a low amount of variance and the significant effects are small.

**FIGURE 2:**
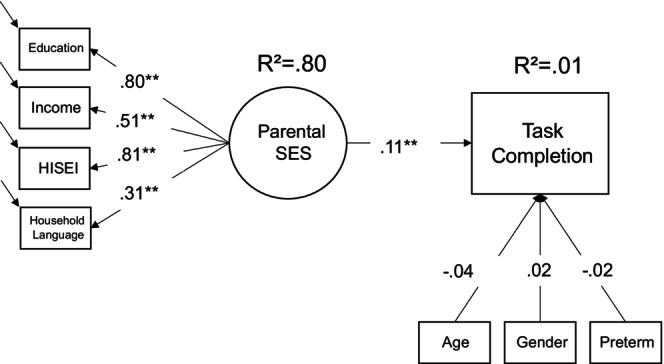
Model predicting completion of the habituation task. *N* = 3129 (FIML), *χ* ^2^(17) = 45.81, *p* < .01. CLI = .988, RMSEA = .02, SRMR = .02 (standardized coefficients reported); **p < .01. Coding: 0 = Task non‐completion; 1 = Task completion.

For the model on task completion, we analysed a subsample with *N* = 3129 cases; *N* = 2945 completed both habituation subtasks. Figure 2 shows that the overall model is comparable to the findings on participation; for a subsample comparison regarding the descriptive information, see Table 2 and Table S1. Parental SES is positively associated with task completion, indicating that in this already selective subsample, children from lower socioeconomic backgrounds are still more likely to drop out during the habituation task. None of the control variables has a significant effect on task completion. Overall, the model explains a low amount of variance and the effect of parental SES is small.

**FIGURE 3:**
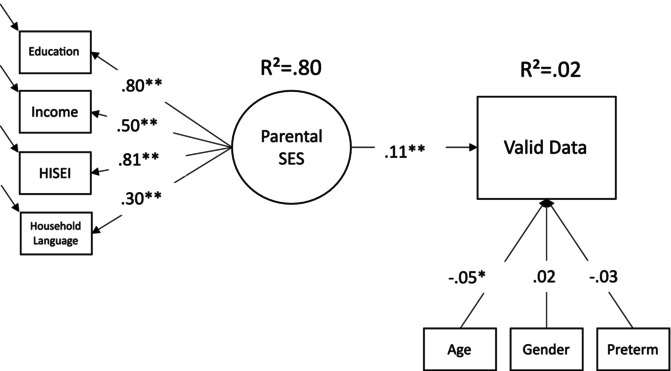
Model predicting valid looking data. *N* = 2945 (FIML), *χ* ^2^(17) = 49.04, *p* < .01. CLI = .985, RMSEA = .03, SRMR = .02 (standardized coefficients reported); *p < .05, **p < .01. Coding: 0 = No valid looking data available; 1 = Valid looking data available.

Finally, we also analysed a stricter subsample, considering only cases in which the video recording could be coded, and actual looking data were released in the scientific use file (*N* = 2945; *N* = 2354 cases with valid looking data). In this exploratory model, the findings mirror the previous analyses (Figure 3). Parental SES is positively associated with available looking data, indicating that even after selectivity due to participation and task completion, children from higher socioeconomic backgrounds are still more likely to have their looking data published. Overall, the exploratory model explains a low amount of variance and the effect of parental SES is small.

## DISCUSSION

Many researchers argue that the sample structure in infant research is important for what kind of results are found and how such results can be interpreted (Bornstein et al., 2013; Oakes, 2017; Singh et al., 2023). Selectivity is a threat to the validity of research findings, and infant studies are often struggling with selective samples because data from this group are hard to collect and behavioural measures are often too complex to implement in large‐scale studies. Therefore, the current paper investigated the effects of infants' socioeconomic background, namely, parental SES, on attrition in a habituation task administered at 7 months in the context of a German large‐scale panel study. Infants' socioeconomic background was significantly associated with attrition in multiple ways: Infants from lower socioeconomic backgrounds were less likely to participate (i.e., their parents were less likely to give informed consent) and complete the habituation task (i.e., have a complete video recording of both subtasks). Furthermore, in an exploratory analysis with a stricter subsample definition, we also found that the videos from children from lower socioeconomic backgrounds were less likely to be codable. Although the effects in all models were small and the explained variance suggests that there should be more substantial predictors (e.g., child temperament and health; time constraints of the parents; Albaum & Smith, 2012), we consider these effects robust due to the heterogeneous data from a large‐scale study. Regarding the effects of the control variables, only age was partially significantly associated with attrition, while gender and preterm birth status were not. Older children were less likely to participate and have codable video recordings probably because they are more active during the habituation task. However, because this effect was small and not significant in all models, we cannot draw firm conclusions about possible age effects.

It should also be mentioned that both participation and task completion are heterogeneous phenomena that cannot be comprehensively investigated with the current data. Regarding participation, the net sample of NEPS SC1 was already moderately biased regarding parental education, migration background and household size (Würbach et al., 2016). In a strict sense, true participation effects could only be investigated with the discrepancy between the gross and net sample (Eysenbach, 2005). Although this was not possible, we argue that the data still include more background information and a more heterogeneous sample than most laboratory studies. Therefore, it is noteworthy that in all subsamples, we still found SES effects. This highlights that although there was already an initial selectivity in each subsample, children's socioeconomic background still predicted attrition. It seems there are multiple barriers associated with participation and task completion. Parents from lower socioeconomic backgrounds may, for example, have a more negative attitude towards scientific research, have less time due to straining working conditions, or struggle with the academic language that is often featured in surveys, which could result in higher perceived costs for participating (Glaser, 2012). In NEPS SC1, the respondents were visited at home and great care was taken regarding the (initial) representativeness of the sample (Zinn et al., 2020), which is why we expect that such barriers may be even higher for typical laboratory research.

Importantly, these findings should be interpreted in the context of the household setting, which is not as standardized as laboratories. This is also evident in the small number of cases that did not complete the habituation task (5.88%) and the high number of cases with non‐valid looking data (20.07%). In other words, participation but not necessarily the data quality could have benefitted from the setting. Thus, the household setting could have resulted in higher rates of participation but also higher rates of task non‐completion due to potential technical or external disturbances (which were not covered by the current analyses). Overall, these findings should be relevant for the interpretation of past studies on social inequality using this data, which probably underestimated selectivity effects (Weinert et al., 2017), as well as future research planned in a household setting.

Infant research in the last few years has shifted towards the household setting, with researchers conducting experiments remotely. This shift originated in the demand for collecting more data from typically under‐represented members of society (Singh et al., 2023) and was reinforced due to the COVID‐19 pandemic. While much of this work is still in early stages, there is a recent meta‐analysis suggesting that the effect sizes between in‐person laboratories and remote testing are not significantly different, even in young children (Chuey et al., 2024). Large‐scale assessments in the ManyBabies project have also indicated that toddlers' anticipatory looking behaviour may be adequately captured using remote eye‐tracking methods, although the attrition rate was about four times as high as in a typical laboratory setting (Steffan et al., 2024). Overall, remote data collection may become a viable way to counter socioeconomic differences in participation and task completion.

In addition to about one‐fifth of the children who are not represented in the available data, it could be that certain aspects of children's socioeconomic background also have an impact on their observable behaviour, such as crowded and noisy households that often go together with low income (Evans et al., 2010). There have been few studies that specifically investigated whether such structural aspects are also associated with the looking behaviour in habituation tasks. Mayes and Bornstein (1995) did not find any significant effect of maternal education on habituation task performance in 5‐month‐old infants. However, a newer study investigated children's homes more directly and found that infants living in noisy environments show impairments in their information processing as measured by habituation tasks (Tomalski et al., 2017). Still, NEPS SC1 does not provide detailed information on whether such issues are prevalent. Another issue that could not be addressed with the current data is the stability of attrition. Previous research reported considerable short‐term stability of non‐completion (38% of all children repeatedly did not complete a habituation task across 2 weeks; Bell et al., 2002). However, more recent research found no systematic stability of task completion between different experimental tasks (Segal et al., 2021). Although NEPS SC1 also featured a habituation task when the children were 17 months old, the survey plan substantially differed compared to when the children were 7 months old (Attig et al., 2023).

While there are different terms to address the phenomenon of attrition, there is no consistency in the literature. Therefore, it is sometimes unclear whether attrition refers to non‐participation or non‐completion, and the reasons for both are also not always differentiated (e.g., internal or external reasons). The present findings showed that there are differential effects of both participation and task completion. Further research could investigate the usage of these terms in a systematic review. The findings imply that aspects of the children's socioeconomic background, namely, maternal education, household income, occupational status and household language, are relevant for different levels of attrition. Interviewer or test administrator characteristics in the context of large‐scale surveys or small‐scale laboratory experiments should be important for countering such effects. Interviewers could, for example, focus on reducing language barriers with translated or symbolic instructions, or improve the way they motivate respondents or show empathy for their situation (Ribisl et al., 1996) – such aspects are often found to be positively related to subject retention in longitudinal designs (Lynn et al., 2014; Ongena & Dijkstra, 2021), especially in face‐to‐face settings, which are still predominant in infant research. Future research could also address data security concerns as the current findings indicate that this was an issue for a substantial amount of people. Although this kind of data is notoriously problematic to collect as people with such concerns usually do not take part in the first place (Holtom et al., 2022), it is reasonable that such concerns can be remedied with a straightforward and transparent explanation of how the data will be processed by the task administrators.

A considerable amount of the research on attrition concerns methodological strategies for reducing attrition due to careful data collection plans (Ribisl et al., 1996) or how to statistically model missing values (Nicholson et al., 2017). Our findings suggest that there are multiple barriers to participating and completing a habituation task associated with children's socioeconomic background. The current large‐scale panel data suggest that more participants would have given informed consent if the experimental setup or privacy concerns were better addressed. This should provide valuable information for the increased efforts in remote testing, which face similar issues. In addition, oversampling, which is a useful if expensive approach (Bornstein et al., 2013), should also consider that attrition affects multiple stages in the data collection. Although some studies indicate that task completion in habituation tasks is probably unstable and may not bias certain results (Klein‐Radukic & Zmyj, 2015; Segal et al., 2021), the current findings imply that sample selectivity in participation and task completion do influence what kind of data are available in the first place.

## AUTHOR CONTRIBUTIONS

**Maximilian Seitz:** Conceptualization; data curation; methodology; writing – original draft; formal analysis. **Dave Möwisch:** Conceptualization; writing – review and editing. **Manja Attig:** Conceptualization; writing – review and editing.

## CONFLICT OF INTEREST STATEMENT

The authors declare that the research was conducted in the absence of any commercial or financial relationships that could be construed as a potential conflict of interest.

## Supporting information


Appendix S1.

## Data Availability

The data that support the findings of this study are openly available in NEPS DATA at https://doi.org/10.5157/NEPS:SC1:8.0.0.
