# Supplementary material for: Attrition in a large‐scale habituation task administered at home
Source: Br J Dev Psychol. 2024 Oct 25;43(1):124–38. doi: 10.1111/bjdp.12528 (PMC11823327; doi:10.1111/bjdp.12528)
Supplement: Supplementary file 1 — Appendix S1. [file BJDP-43-124-s001.docx]

**Supplement**

Task completers were younger than non-completers (*t*(3120) = -3.66, *p* < .01, *d* = 0.15) and less often had preterm birth status (*t*(3127) = -2.26, *p* = .02, *d* = 0.09). Task completers also had mothers with higher levels of education (*t*(3081) = 5.91, *p* < .01, *d* = 0.25), higher income (*t*(3032) = 4.68, *p* < .01, *d* = 0.20), a higher occupation level (*t*(2697) = 5.09, *p* < .01, *d* = 0.23), and were more likely to have German as their main language at home (*t*(3127) = 5.28, *p* < .01, *d* = 0.22).

Table S1

Task Completion: Descriptive Overview of Predictors and Control Variables

|  | Task Completion (*N* = 2,945) | | Task Non-Completion (*N* = 184) | |
| --- | --- | --- | --- | --- |
| Variable | *M* (*SD*) | Range | *M* (*SD*) | Range |
| Education (years) | 14.72 (2.57) | 9-18 | 13.79 (2.81) | 9-18 |
| Household Income (€) | 1,667.23 (901.22) | 95.24-15,555.56 | 1,435.04 (813.28) | 363.64-7,142.86 |
| HISEI | 63.45 (19.54) | 12.01-88.96 | 56.80 (22.06) | 14.21-88.70 |
| Household language | 406 (13.79%) not only German; 2,539 (86.21%) German | | 41 (22.28%) not only German; 143 (77.72%) German | |
| Age (months) | 6.98 (0.73) | 5.77-11.93 | 7.11 (0.92) | 5.77-11.67 |
| Gender | 1,508 male (51.21%); 1,437 female (48.79%) | | 89 male (48.37%); 95 female (51.63%) | |
| Preterm | 2,709 full-term (91.99%); 236 preterm (8.01%) | | 165 full-term (89.67%); 19 preterm (10.33%) | |

*Note*. Missing values: Age (0.22%); education (1.47%); household income (3.04%); HISEI (13.74%); gender (0%), preterm (0%); household language (0%).

The group with valid looking data was younger than the group with no valid looking data (*t*(2937) = -2.89, *p* < .01, *d* = 0.13) and less often had preterm birth status (*t*(2943) = -1.97, *p* = .05, *d* = 0.09). In addition, this group had mothers with higher levels of education (*t*(2900) = 4.42, *p* < .01, *d* = 0.21), higher income (*t*(2852) = 3.56, *p* < .01, *d* = 0.17), a higher occupation level (*t*(2544) = 3.72, *p* < .01, *d* = 0.19), and was more likely to have German as their main language at home (*t*(2943) = 4.49, *p* < .01, *d* = 0.21).

Table S2

Valid Looking Data: Descriptive Overview of Predictors and Control Variables

|  | Valid Looking Data (*N* = 2,354) | | No Valid Looking Data (*N* = 591) | |
| --- | --- | --- | --- | --- |
| Variable | *M* (*SD*) | Range | *M* (*SD*) | Range |
| Education (years) | 14.82 (2.53) | 9-18 | 14.30 (2.71) | 9-18 |
| Household Income (€) | 1,696-94 (869.36) | 120-15,555.56 | 1,546.07 (1012.76) | 95.24-14,285.71 |
| HISEI | 64.16 (19.18) | 12.01-88.96 | 60.55 (20.72) | 14.21-88.96 |
| Household language | 291 (12.36%) not only German; 2,063 (87.64%) German | | 115 (19.46%) not only German; 476 (80.54%) German | |
| Age (months) | 6.96 (0.71) | 5.77-11.93 | 7.05 (0.77) | 5.90-10.69 |
| Gender | 1,219 male (51.78%); 1,135 female (48.22%) | | 289 male (48.90%); 302 female (51.78%) | |
| Preterm | 2,177 full-term (92.48%); 177 preterm (7.52%) | | 532 full-term (90.02%); 59 preterm (9.98%) | |

*Note*. Missing values: Age (0.20%); education (1.46%); household income (3.09%); HISEI (13.55%); gender (0%), preterm (0%); household language (0%).

Table S3

Overview of Bivariate Correlations

|  | Participation (0=no; 1=yes) | 1. | 2. | 3. | 4. | 5. | 6. | 7. | 8. |
| --- | --- | --- | --- | --- | --- | --- | --- | --- | --- |
| 1. Task Completion (0=incomplete; 1=complete) | - |  |  |  |  |  |  |  |  |
| 2. Valid Looking Data (0=invalid; 1=valid) | - | - |  |  |  |  |  |  |  |
| 3. Age (months) | -.07** | -.04* | -.05** |  |  |  |  |  |  |
| 4. Gender (1=male; 2=female) | .00 | -.01 | -.02 | -.01 |  |  |  |  |  |
| 5. Preterm (0=no; 1=yes) | -.03 | -.02 | -.04* | .00 | .02 |  |  |  |  |
| 6. Education (years) | .15** | .08** | .08** | -.03 | .02 | -.03 |  |  |  |
| 7. Household Income (€) | .11** | .06** | .07** | .01 | .00 | -.02 | .41** |  |  |
| 8. HISEI | .15** | .08** | .07** | .00 | .01 | -.05** | .65** | .44** |  |
| 9. Household language (0=not only German; 1=only German) | .11** | .06** | .08** | -.01 | .03 | .02 | .29** | .16** | .24** |

*Note*. Bivariate correlations of all variables used in the models (*N* = 2,699-3,481); Pearson correlations and point-biserial correlations reported. Participation, task completion, and valid looking data cannot be correlated due to collinearity; * *p* < .05, ** *p* <. 01.

Table S4

Habituation Task Stimulus Material (Examples)

|  | Habituation Phase (Example) | Dishabituation Phase (Example) |
| --- | --- | --- |
| First Subtask (Categorical Flowers) | 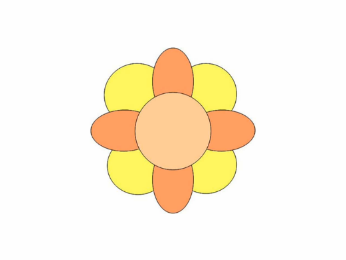 | 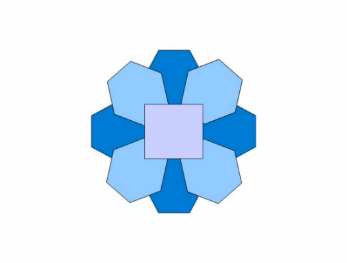 |
| Second Subtask (Categorical Bugs) | 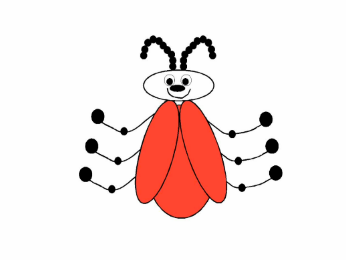 | 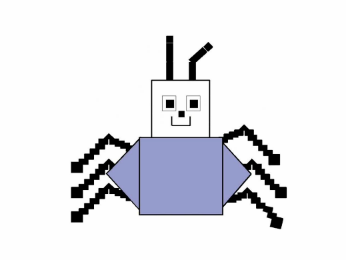 |

*Note*. The categorical flowers were adapted from similar material used at the University of Heidelberg, Germany (Pahnke, 2007); the categorical bugs were tested and used at the University of Bamberg, Germany (Zhang, 2007). The complete stimulus material can be found in the official documentation (Seitz et al., 2023).

**References**

Seitz, M., Attig, M., Möwisch, D., & Weinert, S. (2023). *Visual habituation-dishabituation tasks in NEPS Starting Cohort 1: Approaches to interpreting the data (NEPS Survey Paper No. 102)*. Leibniz Institute for Educational Trajectories. https://doi.org/10.5157/NEPS:SP102:2.0

Pahnke, J. (2007). *Visuelle Habituation und Dishabituation als Maße kognitiver Fähigkeiten im Säuglingsalter. Individuelle Differenzen in Habituationsaufgaben zur Unterscheidung einzelner vs. kategorialer Stimuli* *[Visual habituation and dishabituation as indicators of cognitive abilities in infancy. Individual differences in the discrimination of individual vs. categorial stimuli in habituation tasks]* [Doctoral dissertation, University of Heidelberg, Germany]. http://www.ub.uni-heidelberg.de/archiv/7459

Zhang, D. (2007). *Learn a word learning constraint: Emergence of the taxonomic constraint and its relationship with early word acquisition* [Doctoral dissertation, University of Bamberg, Germany]. https://fis.uni-bamberg.de/handle/uniba/125
